# Should researchers use single indicators, best indicators, or multiple indicators in structural equation models?

**DOI:** 10.1186/1471-2288-12-159

**Published:** 2012-10-22

**Authors:** Leslie A Hayduk, Levente Littvay

**Affiliations:** 1 Department of Sociology, University of Alberta, Edmonton, Alberta, T6G 2H4, Canada; 2 Department of Political Science, Central European University, Nador u. 9, Budapest, H-1051, Hungary

**Keywords:** Single indicators, Factor analysis, Multiple indicators, Testing, Structural equation model

## Abstract

**Background:**

Structural equation modeling developed as a statistical melding of path analysis and factor analysis that obscured a fundamental tension between a factor preference for multiple indicators and path modeling’s openness to fewer indicators.

**Discussion:**

Multiple indicators hamper theory by unnecessarily restricting the number of modeled latents. Using the few best indicators – possibly even the single best indicator of each latent – encourages development of theoretically sophisticated models. Additional latent variables permit stronger statistical control of potential confounders, and encourage detailed investigation of mediating causal mechanisms.

**Summary:**

We recommend the use of the few best indicators. One or two indicators are often sufficient, but three indicators may occasionally be helpful. More than three indicators are rarely warranted because additional redundant indicators provide less research benefit than single indicators of additional latent variables. Scales created from multiple indicators can introduce additional problems, and are prone to being less desirable than either single or multiple indicators.

## Background

Structural equation modeling melds path analysis and factor analysis under a common statistical framework. The multiple-indicator factor tradition includes works by Thurstone [1], Harman [2], Lawley & Maxwell [3], and Mulaik [4], while the single-indicator path tradition has roots in regression and includes Wright [5,6], Blalock [7], Duncan [8], and Heise [9]. Recent structural equation introductions range from having a heavy factor focus (Byrne [10]), through works seemly oblivious to path-factor tensions (Kline [11], Byrne [12,13], Bollen [14]), to path oriented discussions (Hayduk [15,16]). The path and factor approaches differ noticeably in regard to procedure, testing, and indicators.

In arguing against Anderson & Gerbing’s [17,18] procedural suggestion to use a factor model before introducing latent paths, Fornell and Yi [19,20] implicitly contrasted the path and factor approaches. Hayduk’s [16] additional critiques of the factor-model-before-path-model idea led to extensive SEMNET [21] discussions and a special issue of *Structural Equation Modeling* where a target article challenging the use of factor-models before latent path-models (Hayduk and Glaser [22]) was followed by commentaries (Mulaik and Millsap [23], Bollen [24], Bentler [25], Herting & Costner [26]), and a rejoinder (Hayduk and Glaser [27]). The weaknesses of the factor-model-first idea became painfully obvious, so subsequent SEMNET discussions switched to the topic of model testing – which again pitted the path-model inclined (who favored diagnostic attention to significant evidence of model ill-fit) against the factor-model inclined (who sought to replace model testing with indexing). This led to a special issue of *Personality and Individual Differences* in which Barrett’s [28] target article called for reporting and respecting the model χ^2^ test. Barrett’s call was neither strong nor precise enough for some (Hayduk, Cummings, Boadu, Pazderak-Robinson, & Boulianne [29], McIntosh [30]) but was “challenging” to those having factor analytic backgrounds (Millsap [31], Mulaik [32], Steiger [33]) – though the disarray among the dissenting replies signaled that careful model testing constitutes the new norm, even for factor models.

One additional path-versus-factor battle awaited, namely the matter of latents having single indicators (Hayduk & Pazderka-Robinson [34], Hayduk [16]). SEMNET again hosted multiple skirmishes, but it fell to the current article to organize the arguments regarding latents having relatively few indicators.

### Organizing the issues

**Figure 1:**
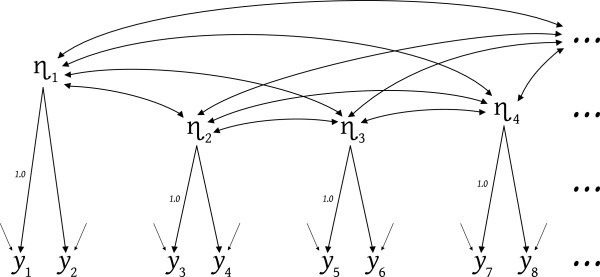
Two indicators per latent.

We begin with the Figure 1 model which has two indicators per latent variable – not the multiple indicators requested by factor models but also not single indicators. This figure emulates LISREL notation (Joreskog & Sorbom [35]) where η’s are true-score-like latent variables and *y*’s are indicator variables, but this model is not complete – as indicated by the dots representing "the rest of the model". The paired indicators report that the author of Figure 1 attended to the measurement methodology distinguishing each indicator pair from the other pairs (e.g. questionnaire wordings). The indicator pairings also signal that the researcher is not doing exploratory factor analysis because exploratory factor analysis is not likely to locate half as many latents as indicators, or indicators clustered in tidy pairs. 

This model contains a strange conceptual bifurcation. The model claims considerable causal understanding in one model segment (the latents’ effects on the indicators) and complete causal ignorance in another segment (the saturated non-directional relationships among the latents). The researcher constructing this model did not fear causation itself because the model requires latent to indicator causal actions. It is more likely that the causal-segmentation arose from the complexity and difficulty of considering specific latent-to-latent causal connections. It is common to not know the latent level causal structure. But how should a structural equation modeler proceed when they don't know the latent causal structure?

**Figure 2:**
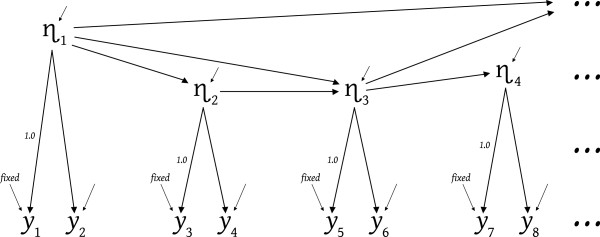
Incorporating latent causal structuring.

Researchers following factor analytic tradition were trained to think it was OK to specify measurement structures before introducing latent effects and constraints. The deficiencies of the measurement-before-latent-structure idea were headlined in Hayduk & Glaser [22,27], Hayduk [16], and Fornell & Yi [19,20], so we need not revisit these details here. Let us instead presume the researcher encountered theory-encouraging training that overcame their causal-segmentism, and postulated the latent causal structure depicted in Figure 2. This particular battle has been won whether the postulated structure is correct or not, because the battle was to get the researcher to see, understand, and incorporate some reasonable (to them) theoretical causal structuring, to permit the indicator data to speak for or against the researcher’s theory/thinking. There is an undeniable preference for the data speaking approvingly, but theory is furthered whatever the data’s verdict. 

What is required to move from a model like Figure 1 toward a Figure 2 model? One obvious, and difficult, concern is that any postulated latent-level effects should have worldly counterparts, and postulated absences of effects should correspond to worldly lacuna. A less obvious but important concern is that each latent variable participating in latent-to-latent causal effects must be identical to the latent acting causally toward specific indicators. This requirement actually provides substantial assistance, as we see shortly. We address the fixed measurement error variances depicted in Figure 2, later in this article. When we refer to measurement error variances, true scores, and the like, our concern is for validity via models that match the relevant worldly causal structures, rather than the mere reliability connotations such terms can carry in the context of classical test theory.

Consider a variable like η_3_ that is somewhere in the midst of the effects among the latents. η_3_'s value (for any one case) is set by absorbing diversity (absorbing the potentially very different styles and magnitudes of effects arriving from η_1_, η_2_, and η_3_'s error variable), and η_3_ emits the resultant value proportionately – namely in proportion to the magnitudes of the effects η_3_ emits. To specify such a causal nexus for η_3_ the researcher must theorize or understand η_3_ as being capable of the relevant absorptions and emissions – including η_3_'s causal effects on its indicators.

Consider the causal connection between η_3_ and y5. The 1.0 effect depicted in Figure 2 does not make y5 a perfect reflection of η_3_ – it merely asserts a scale for η_3_ by asserting that each "perfect and isolated unit increase" (or decrease) in the true value of η_3_ (whether originating in η_1_, η_2,_ or η_3_'s error) would result in a corresponding unit increase (or decrease) in the indicator’s scaled value. This isolation and perfection is imaginary because a real unit change in latent η_3_ would mix with the measurement-error forces that also pummel the observed value of y5. The measurement error effects would nudge y5's value to be somewhat more or less than the perfect unit change originating in η_3_.

#### Error variances and latent meanings

The variance of the error-5 variable connected to y5 helps determine the meaning of the latent variable η_3_. If there were no causal variables influencing y5 other than η_3_, there would be no variance in the error-5 variable, and y5’s observed values would correspond exactly to, and have the same variance as, the true values of η_3._ Seeing y5’s values would directly report η_3_’s true values.

An opposite extreme occurs if the causal variables collectively referred to as y5’s error variable produce most of the variability in y5's values. Each real unit change in η_3_ still produces a corresponding unit for unit change in y5, but if the causal actions of the variables cumulated as y5’s error variable knock y5's values hither and yon, what is the identity of the η_3_ variable? η_3_ becomes any one of the many potential things that produces a minor amount of variation in y5's values. η_3_'s identity is thrown into doubt because it becomes one (an unknown one) of the variables capable of producing some of y5’s variance.

Let us consider the more realistic case where η_3_ is neither perfectly reflected in y5's values, nor so minimally contributing to y5 that the researcher should consider discarding y5. The researcher presumably scaled η_3_ via the 1.0 effect to y5 because y5 was the best available indicator. For example, if y5 came from questionnaire data: the question providing y5 presumably was clear, precise and appropriately worded, there were few missing values, no recoding difficulties, no socially-desirable response, a reasonable distribution across multiple evenly-spaced response options, and so on. Being the best of the available indicators makes y5 unlikely to be almost all error, but it is also unlikely to be error-free.

**Figure 3:**
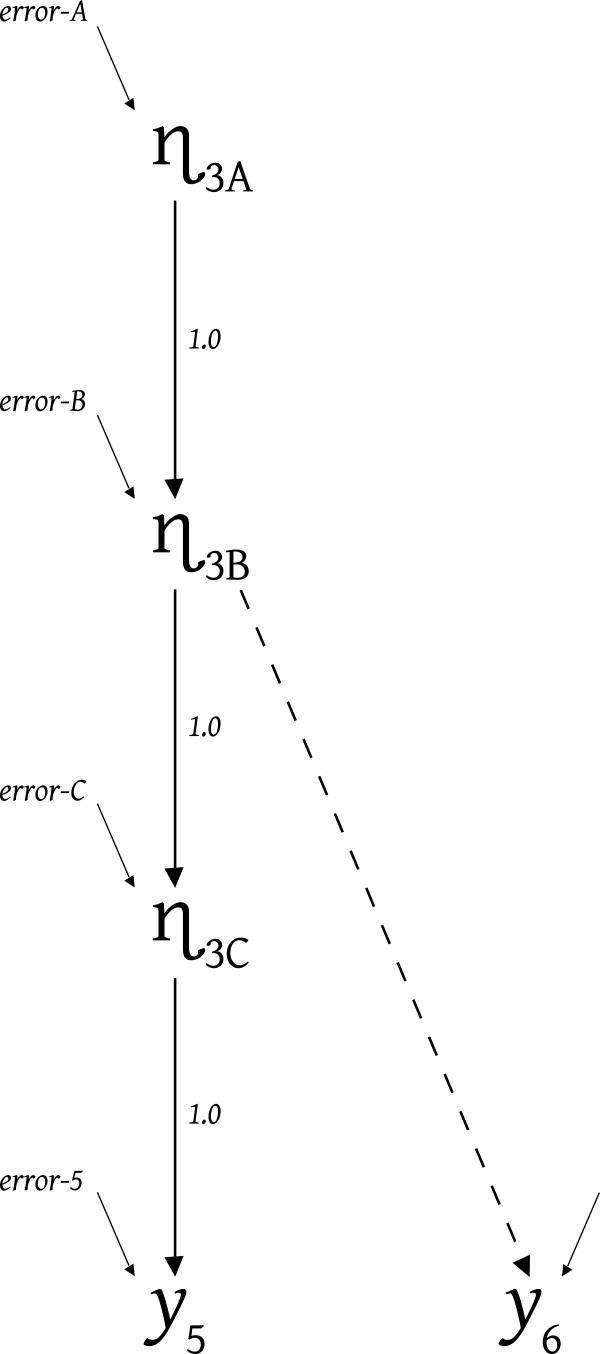
**Clarifying η**_**3**_.

The questionnaire or measuring instrument is insufficient to dictate what constitutes measurement error in a variable like y5 because the latent-effect portion of the model contributes importantly to η_3_’s identity or meaning. Only causes of y5 other than η_3_ constitute error. Figure 3 illustrates three options for what η_3_ might be, namely: η_3A_, η_3B_ or η_3C_. Any of these three latent variables could be the η_3_ latent measured by y5 in Figure 2 because all three of these latents cause y5. Momentarily ignore the dashed effect leading to y6, and notice that if η_3C_ was the intended identity of η_3_ only the real causal variables summarized as error-5 would provide measurement error. The causal features subsumed within error-C would produce variations in the true values of η_3C_ and subsequently true-score (not error) variance in y5. But if η_3B_ was the intended identity of η_3_, the error on y5 in Figure 2 would be the sum of error-5 and error-C from Figure 3. The variables whose causal impacts constitute the “errors” entering at both η_3C_ and y5 in Figure 3 would tend to obscure how the true values of η_3B_ would make themselves apparent in y5's values. The error on y5 in Figure 2 is the cumulated, or net, effect of all the causal impacts entering anywhere along the causal chain leading from the intended latent variable, here η_3B_, to y5, and not just effects impinging directly onto y5.

Similarly, if η_3A_ was the intended meaning for η_3_ in Figure 2, then all the “error” sources impinging upon the chain of indirect effects between η_3A_ and y5 would constitute disruptive causal forces obscuring the true value of η_3A_. Hence, the error on y5 in Figure 2 would be the sum or accumulation of the causal features labeled error-B, error-C, and error-5 in Figure 3. While it is common to label disruptive causal forces connected to latents as residuals or structural-disturbances, and as measurement-errors if connected to indicators, we label all these as “errors” in Figure 3 because which specific disruptive causal forces constitute measurement-errors and which “residuals” remains open and requires careful researcher assessment.

The chain of effects leading through the three optional η_3_'s to y5 warrants the use of y5 as an indicator of any one of η_3A_, η_3B_, or η_3C_, and the choice of which of these the researcher intends to incorporate in the latent level of the model dictates which causal actions constitute disruptions that should be accumulated into y5's error variable in Figure 2. The more extensive the disruptive forces, the larger the proportion of y5’s variance that is error but it is important to remember that the fundamental issue concerns the validity of the latent’s specification and not mere reliability.

Now we reverse the statements about error accumulation. By specifying the appropriate error-accumulation (the appropriate portion of y5’s variance) as fixed, the researcher could select whether the Figure 2 model contains η_3C_ or η_3B_ or η_3A_. To use η_3C_ (in Figure 2) fix the variance of the error on y5 in Figure 2 to be the variance provided by only error-5. To select η_3B_, the variance on y5's error in Figure 2 should include variance produced by both error-5 and error-C in Figure 3. And if η_3A_ is the appropriate η_3_ for inclusion in Figure 2, the error on y5 would arise from error-5, error-C, and error-B in Figure 3. Specifying the portion of the variance of y5 that arises from "error disruption" selects whether η_3A_ or η_3B_ or η_3C_ is the variable the researcher views as contributing the complementary true-score portion of y5's variance.

The mathematical foundation for distinguishing between η_3A_, η_3B_, and η_3C_ on the basis of the proportion of y5’s variance that is error is straight forward. For the Figure 3 model

(1) $$y5=\eta_{3C}+error5$$

Assuming the independence of the error variables from one another and from the causally preceding η’s, this implies.

(2) $$Var\left(y5\right)=Var\left(\eta_{3C}\right)+\left\{Var\left(error5\right)\right\}$$

In Figure 3*η*_3*C*_ = *η*_3*B*_ + *errorC* and inserting this into Equation-1 says

(3) $$y5=\eta_{3B}+errorC+error5$$

which implies

(4) $$Var\left(y5\right)=Var\left(\;\eta_{3B}\right)+\left\{Var\left(errorC\right)+Var\left(error5\right)\right\}$$

And similarly, inserting *η*_3*B*_ = *η*_3*A*_ + *errorB* into Equation-3 provides

(5) $$y5=\eta_{3A}+errorB+errorC+error5$$

which implies

(6) $$Var\left(y5\right)=Var\left(\;\eta_{3A}\right)+\left\{Var\left(errorB\right)+Var\left(errorC\right)+Var\left(error5\right)\right\}$$

The variance of indicator y5 is partitioned by the Figure 3 causal world, and Equations 2, 4, and 6 illustrate how any one of the latent variables η_3C_, η_3B_, or η_3A_ could be validly introduced as η_3_ in Figure 2 by fixing y5’s error variance at the sum of the appropriate error variances presented within braces above.

#### A second indicator and potential incompatibility

To determine whether η_3A_, η_3B_, or η_3C_ is validly used in the Figure 2 model, the researcher must consider more than just the identity and causal termini of disruptive “error” variables. They must also consider any additional available indicators such as y6. Figure 3 depicts η_3A_ as an indirect cause of y6, η_3B_ as a direct cause of y6, and η_3C_ as not causing y6. Consider what would go wrong "statistically" (actually model implicationally) if η_3_ in Figure 2 was called, or given a “meaning,” corresponding to either η_3A_ or η_3C_ when in fact η_3B_ was the direct causal source of y6. That is, consider the model implications, or model claims, that go awry if η_3_ (in Figure 2) were mis-identified as η_3A_ or η_3C_ because y6 was directly caused by η_3B_ (as in Figure 3).

This requires that we attend to how a common-cause implies, or causally produces, a spurious covariance or correlation between two variables. If the value of a common cause increases, the values of both the effected variables increase (presuming positive effects). If the value of the common cause decreases, the values of both effected variables decrease. Hence the values of the effected variables become coordinated (both tending to rise or fall together). The extent of the coordination or covariation depends on the strengths of the two causal effects, and on the variability in the values of the common cause. Considering Bollen ( [14] page 22), Duncan ( [8] page 14), or Hayduk ( [15] page 31; [16] pages xvi,10) will convince you that the covariance between two variables effected by a common cause must equal the product of the two effects and the variance of that common cause. Specifically, for a common cause of y5 and y6, this requires that

(7) $$Cov\left(y5,y6\right)=\left(effect\;leading\;to\;y5\right)\left(effect\;leading\;to\;y6\right)\left(variance\;of\;the\;common\;cause\right)$$

Consider what this equation implies if y6 (in Figure 2) was thought of as having common cause η_3A_, or η_3B_ (as diagramed in Figure 3), or η_3C_. In all three instances, the effect leading to y5 would be 1.0 – whether a 1.0 direct effect, or an indirect effect of 1.0 obtained from the product of several 1.0 effects. This constitutes a way of providing the latent variable (whether η_3A_, η_3B_, or η_3C_) a scale that corresponds to y5’s scale units. If η_3C_ was the common cause in Figure 2, the model-required covariance between the y5 and y6 indicators (from Equation 7) would be

(8) $$Cov\left(y5,y6\right)=\left(1.0\right)\left(the\;effect\;of\;\eta_{3C}\;on\;y6\right)\left(variance\;of\;\eta_{3C}\right)$$

And if η_3B_ was the common cause (as depicted in Figure 3) the model-implied covariance between the indicators would be

(9) $$Cov\left(y5,y6\right)=\left(1.0\right)\left(the\;effect\;of\;\eta_{3B}\;on\;y6\right)\left(variance\;of\;\eta_{3B}\right)$$

and if η_3A_ was the common cause in Figure 2 the model-implied or model-required covariance would be

(10) $$Cov\left(y5,y6\right)=\left(1.0\right)\left(the\;effect\;of\;\eta_{3A}\;on\;y6\right)\left(variance\;of\;\eta_{3A}\right)$$

The covariance on the left of these equations is what the Figure 2 model, with its common-cause structure and effect magnitudes, implies should be observed as the covariance between y5 and y6 for the three optional meanings for η_3_. Naturally, since we are seeking a valid model, we hope the model’s implication matches the observed data covariance between y5 and y6.

Now return to Figure 3 and notice that the variances of variables η_3A_, η_3B_ and η_3C_ differ; with η_3A_ having the smallest variance and η_3C_ the largest variance because the variance-producing causal actions of additional “error” variables impinge on the chain of latent variables in moving from η_3A_ toward η_3C_. Any of these different latent variances, when placed on the right sides of Equations 8, 9, or 10, could imply a covariance (on the left of the equation) that matches the observed y5 y6 covariance by making a compensating adjustment to the “estimated” magnitude of the effect leading from each latent-option to y6. The latent with the largest variance (η_3C_), could be given the weakest estimated effect leading to y6 to make the product of the entries on the right of Equation 8 correspond to the observed Cov(y5,y6), and so forth.

Hence, altering whether we choose η_3A_, η_3B_, or η_3C_ to be the η_3_ to use in the Figure 2 model would control the magnitude of the “estimated” effect leading to y6 that would match the data covariance between y5 and y6. But only one of the causal connections would be valid in the sense of matching the world's causal structure (η_3B_ if Figure 3 depicts the true causal structure) even though the other optional latents (η_3A_ and η_3C_) could be made to match the covariance between the y5 and y6 indicators via compensating (but incorrect or “biased”) estimates of the effect leading from the selected latent to y6.

No estimate bias would arise if Figure 2 presented y6’s proper causal source, and we specified y5’s error variance as the sum of the error-5 and error-C (from Figure 3) because that selects η_3B_ and implies use of Equation 9, which in turn results in an appropriate estimate for η_3B_’s effect on y6. But selecting either η_3A_ or η_3C_ to appear in Figure 2 (via accumulation of more or fewer errors in Figure 3) would result in an incorrect (biased) estimate of the effect of η_3_ on y6. η_3A_ has too little variance to match the data Cov(y5,y6) with the proper size of effect, and η_3C_ has too much variance to match Cov(y5,y6) with a proper size of effect. In fact, if Figure 3 constitutes the proper causal structure, η_3C_ has no causal effect on y6, and any estimate other than zero is a biased estimate.

#### Latent theory and potential incompatibility

The effect leading from η_3_ to y6 contributes to producing and accounting for many additional data covariances. A zero η_3_ to y6 effect would causally disconnected y6 from all the other model indicators in Figure 2, and hence y6 would display zero covariance with all those indicators. A stronger η_3_ to y6 effect would imply stronger y6 covariances with the indicators of all the causes and effects of η_3_, not just with y5.

The latent-level effects leading to and from η_3_ in the Figure 2 model also depend upon η_3_ having a specific identity – whether η_3A_, η_3B_, or η_3C_. According to Figure 3, η_3B_ is the appropriate version of η_3_ for matching the covariance between y5 and y6, but we have not yet confirmed that η_3B_ is also the version of η_3_ required to engage in causal actions at the latent level of the Figure 2 model – where η_3_ receives effects from η_1_ and η_2_, and sends effects to η_4_ and beyond. In Figure 2, η_3A_ might be required as the causal mechanism carrying effects from η_1_ and η_2_ toward the causally down-stream latents (and their indicators), and η_3A_ might also be the version of η_3_ required to act as a common cause coordinating causally down-stream latents (and their indicators). Thus the latent level causal actions might call for η_3A_ (in Figure 2) with its lower variance and (biasedly) stronger effect to y6 (via Equation 10), while the covariance between y5 and y6 calls for latent η_3B_ with its higher variance and weaker effect leading to y6 (via Equation 9). Such inconsistencies constitute model misspecification and result in invalid models, biased estimates, and model ill fit. Hence both the latent-to-latent effects (as in Figure 2) and the single/multiple indicator options (as in Figure 3) must be assessed simultaneously in deciding which meaning of a latent (like η_3_) is appropriate for inclusion in the model. Similarly detailed assessments should accompany each fixed measurement error variance in the model (e.g. for y_1_, y_3_, etc. in Figure 2).

**Figure 4:**
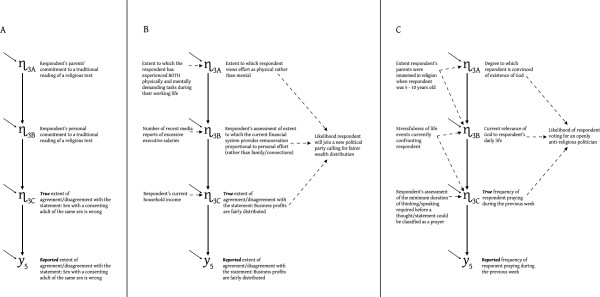
**Hypothetical examples differentiating between η**_**3A**_**, η**_**3B**_**, and η**_**3C**_.

Figure 4 presents hypothetical examples illustrating the kinds of substantive issues a researcher must attend to in the context of difficult attitudinal indicators. In Figure 4A, the causal forces differentiating between the *reported* y5 from the *true* score η_3C_ are things like mistaken recording of a respondent’s verbal response, or the rounding-error implicit when a truly continuous variable is tapped by categoric Likert responses. In contrast, the differences between η_3A_, η_3B_, or η_3C_ reflect substantively different concepts that are progressively causally removed from the specific y5 question wording. The y5 question in Figure 4A neither selects nor forbids any of the three latent meaning/identity options, so the selection from among these depends on the latent-level theory in which the Figure 4A latent is to be embedded.

Figures 4B and 4C are similarly structured to display optional latent-variable identities corresponding to specific indicators, where dashed arrows indicate the kinds of latent-to-latent causal actions a researcher should consider in differentiating between the latent-identities η_3A_, η_3B_, and η_3C_. The selected latent identity might reflect a common disciplinary perspective (e.g, η_3C_ in Figure 4B) but the selection should express the researcher’s theoretical preference and the availability of indicators of the other postulated latent causes/effects, rather than consensus. For example, if the Figure 4C data set had no indicator of “minimum duration for prayer” (or other variables influencing η_3C_), and if the researcher believed no effects led from η_3C_ to downstream latent variables, the researcher would be pushed toward using η_3B_ – namely toward a latent-identity assigning the variance in y5 produced by “minimum duration” (or other η_3C_ causes) as error variance, not true latent variance. That is, attaining a *valid* model specification might require specifying y5 as containing a greater proportion of “error”.

Now back to Figure 3. If η_3A_ is required to appropriately model the covariances of both y5 and y6 with the indicators of the *other* modeled latents, while η_3B_ is required to model the covariance between y5 and y6, the estimation process will attempt to locate compromise estimates for η_3_’s effect on y6, and the effects connecting η_3_ to the other latents. Those compromise estimates can nonetheless result in noticeable inconsistencies between the covariance data and the model’s covariance implications. Such potential inconsistencies render the model testable, but before we turn to testing, let us reconsider the latent level of the model.

#### A new beginning: single indicators as encouraging precision in latent theory

Presuming Figure 3 depicts the true causal structure connecting y5 and y6, we could include both η_3B_ and η_3C_ as latents with single indicators in the model. We do *not* have to choose just one of η_3A_, η_3B_, or η_3C_. We could use y5 as an indicator of η_3C_, and y6 as an indicator of η_3B_, and add precision to the latent level of the model by assessing whether the latent-level effects connected to η_3_ in Figure 2 enter into, or emerge from, specifically η_3B_, or η_3C_, or both. For example, the effect from η_1_ might enter at η_3B_ while the effect from η_2_ enters at η_3C_, and effects might emerge from either η_3B_ or η_3C_ or both on their way to causally-downstream latent variables. Careful consideration of the relevant latent-to-latent effects would be required, and error-C and error-B constitute important parts of the consideration. The variables comprising error-C will have no impact on any latents (other than η_3C_) unless η_3C_ causes some other latent(s) in the model. In contrast, the real variables constituting error-B would influence whatever other latents were caused by either η_3B_ or η_3C_.

If Figure 3 provides the proper causal specification for y5 and y6, using both y5 and y6 as single indicators would *not* permit incorporating both η_3A_ and η_3B_ in the latent level of the model. Either y5 or y6 alone could be used as a valid single indicator of η_3A_ because η_3A_ causes both, and the “intervening” error variables are presumed to be statistically independent, so there is an appropriate style of error accumulation that could be used. But the "other" indicator (whether y6 or y5) could not be used simultaneously as a direct indicator of η_3B_ without misspecifying the causal actions of the variables constituting error-B. For y5 to be an indicator of η_3A_, the real causal actions constituting error-B would have to be part of what is cumulated into y5's error. For y6 to simultaneously be an indicator of η_3B_, those same real error-B sources would have to contribute true variance in η_3B_ – which is contradictory because these real error-B sources cannot be both sequestered as measurement errors dead-ending in y5 and simultaneously be variables causing true-score variance in latent η_3B_ and any variables causally down-stream from η_3B_.

y5 *by itself* would permit incorporating any one of η_3A_, η_3B_, or η_3C_ in the latent level of the model (with error independence and the appropriate accumulation of errors as discussed above), and y6 *by itself* would permit inclusion of η_3A_ or η_3B_ in the model (with appropriate error accumulation). Using y5 and y6 as two single-indicators would permit use of both η_3B_ and η_3C_ in the model but *not* η_3A_ and η_3B_. Using both y5 and y6 as multiple indicators of a single latent would permit *only* η_3B_ in the latent level model (not η_3A_ or η_3C_). y6 could never be justifiably used as an indicator of η_3C_ in any model (even though y6 correlates with η_3C_ due to common cause η_3B_) because the variables constituting error-C do not cause y6. With the causal structuring in Figure 3, using y5 and y6 as multiple indicators of a latent would demand use of η_3B_ at the latent level in Figure 2, whereas use of a single indicator, or a pair of single indicators, would permit the latent level of the model to contain η_3A_ alone, or η_3B_ alone, or η_3C_ alone, or both η_3B_ and η_3C_.

Just as η_3B_ is demanded by modeling y5 and y6 as two indicators of a single latent, multiple indicators in factor analysis demand indicator-controlled latent identities with minimal attention to whether or not the selected latent is capable of appropriate latent-to-latent causal actions. Factor analysis, and scales created from factor-based analyses, force a data-controlled identity onto latents like η_3B_ while disregarding, or even disrespecting, theoretical concern for whether η_3A_, η_3B_, or η_3C_ is, or are, required for appropriate latent level effects. The specification of η_3_ (in Figure 2) as η_3A_, η_3B_, or η_3C_ should not be thought of as being under the exclusive control of η_3_'s indicators. η_3_'s identity is also tied to its latent effects (and absences of effects). The researcher should acknowledge the potential conflict between the latent-level and indicator-level identifications/meanings for η_3_ and preemptively attend to this by holistically assessing both η_3_'s latent level effects and the indicators’ methodology (instrumentation, wordings, scaling, etc.). These observations illustrate why it is preferable to estimate a single full structural equation model rather than attempting to do measurement prior to incorporating latent level effects (as discussed in the 7(1) issue of *Structural Equation Modeling*). The detailed latent considerations prompted by consideration of single indicators should enhance the precision and research contribution provided by structural equation models.

A fixed 1.0 “loading” and a fixed measurement error variance are sufficient to statistically-identify the latent but the larger the fixed measurement error variance the less precise the meaningful-identity provided by a lone indicator. As the specified measurement error variance increases, the latent’s identity is loosened because the latent could be any latent capable of accounting for a decreased proportion of the indicator’s variance. Consequently, latent-level model constraints take stronger control of the latent’s identify with larger measurement error variance specifications. The saturated latent covariances for η_3_ in Figure 1 hamper specification of a consistent latent-and-indicator based identity for η_3_ because the absence of specific required and forbidden latent-level causal connections impedes meaningful differentiation between η_3A_, η_3B_ and η_3C_. A factor analytic claim that η_3_ displays unspecified correlations with other latent factors is too imprecise (too unconstraining) to contribute substantively to identifying η_3_.

As the researcher attends to η_3_'s required (hopefully few) and forbidden (usefully many) latent causes and effects, η_3_'s identity solidifies in the researcher's understanding. That clarified understanding contributes importantly to assessing the strengths and weaknesses of whatever indicators are vying for designation as the “best indicator” because this focuses attention on the specific variables constituting the errors like error-5, error-C, and error-B. Some causes of the optional latent identities might be slated to appear in the model (like η_1_ and η_2_ in Figure 2), and that contributes importantly to deciding whether the required latent is η_3A_, η_3B_, or η_3C_. Assessing which variables’ causal impacts do, or do not, enter between the η_3_ true scores and the indicators’ values clarifies what constitutes measurement error. Researchers may end up disagreeing over the latent’s preferred identity but this constitutes research advancement because it clarifies disagreements previously obscured by conceptual imprecision.

Once the meaning or identity of each latent corresponds to the researcher’s current theoretical understandings, the researcher faces the challenge of getting the model to comply with those understandings so that when data speak about the model they also speak directly about the researcher’s understandings. Most researchers are comfortable incorporating theory assertions about latent effects and absences of effects (as in Figure 2) but researchers should be equally comfortable making measurement error variance assertions because measurement assertions *are* a type of theory assertion.

An effective procedure for maintaining intended theoretical latent meanings was developed decades ago (e.g. Entwisle, Hayduk & Reilly [36]), and was illustrated in Hayduk [15], and summarized in Hayduk [16]. Hayduk’s procedure, as it was dubbed on SEMNET, requires specifying a fixed non-zero measurement error variance for each indicator receiving a 1.0 effect/loading. Fixed measurement error variances are thus provided for all single-indicators, and for the best indicator within each set of multiple indicators. (The other indicators in multiple indicator sets are typically given free loadings and measurement error variances.) The fixed 1.0 provides a scale for the latent and the fixed measurement error variance selects from alternative latent meanings, as in Figures 3 and 4. But before we consider the practical details of fixing measurement error variances, we should consider the statistical identification of the model, and model testing. The fixed measurement error variance procedure we recommend constitutes neither the minimum requirement for model identification nor excessive measurement assertiveness.

#### Identification, testing, and what is tested

If, as in Figure 1, there were four latents and hence eight indicators in the model, there would be 8(8+1)/2 = 36 indicator variances and covariances as data points. The estimated model coefficients would include: 4(4+1)/2 or 10 variances and covariances of the latents, four "loadings" (the other four being fixed 1.0’s scaling the latents), and eight measurement error variances – for a total of 22 estimates. Barring empirical underidentification (which presumes there are no entirely redundant indicators, no entirely disconnected latents or indicators, or indicators having zero variance) these 22 model coefficients should be estimable, and the Figure 1 model should provide a mode χ^2^ test having 36 - 22 = 14 degrees of freedom.

The Figure 2 model would be more assuredly identified than the Figure 1 model, again barring empirical underidentification, which now also presumes no new identification concerns for reciprocal latent effects, loops, excessive latent error covariances, and the like. If there are two fewer effects between the latents than there are covariances among the Figure 1 latents, the Figure 2 model has two more degrees of freedom than the Figure 1 model. And if a fixed measurement error variance is specified for the best of each pair of indicators, this contributes four additional degrees of freedom, making a total of 20 degrees of freedom for the Figure 2 model χ^2^ test. Fixed measurement error variances may be needed to statistically identify some models, but that is not why we need them in the current context, or recommend them in general. The fundamental justification is that fixed measurement error variances clarify the modeled theory, and hence improve the investigation and testing of theory.

Unfortunately, there is no thorough, accurate, and easy specification of what either the Figure 1 or Figure 2 model tests test. It is not as simple as saying Figure 1 tests whether there is a latent underlying each pair of indicators, while a χ^2^–difference test (created as the difference in χ^2^ values and difference in degrees of freedom between the Figure 1 and Figure 2 models) tests whether the postulated latent effects and asserted measurement error variances are correct. These claims are stifled by the possibility that the absence of latent level and measurement error variance constraints permit the Figure 1 model to contain inappropriate compromise latents. Remember that with y5 and y6 as multiple indicators it is impossible to have either η_3A_ or η_3C_ as the η_3_ latent in Figure 1. The absence of specified latent causal constraints on η_3_ in Figure 1 makes it comparatively easy for the Figure 1 model to estimate η_3_ as being η_3B_ (to match the y5- y6 covariance) even if latent η_3A_ was required to match η_3_’s latent-level causal actions. The more stringent latent-level causal requirements on η_3_ in the Figure 2 model make it more difficult for the estimation process to match the data covariances with an inconsistent η_3_ identity. A model requiring η_3A_ will tend to fail even if the appropriate latent-level causal connections for η_3A_ are specified in the Figure 2 model because the covariance between y5 and y6 requires η_3B_. The presence of both y5 and y6 as multiple-indicators pushes for use of η_3B_ in both the Figure 1 and Figure 2 models, but the inconsistency of this forced use of η_3B_ (when η_3A_ is required) is less detectable in Figure 1. The more specific and more demanding latent causal constraints on η_3_ in Figure 2 make it easier to detect the inconsistency between one part of the model (the latent level) requiring η_3A_ with its smaller variance, while another part of the model (the latent common cause of y5 and y6) requires η_3B_ with its larger variance. The Figure 1 model has sufficient degrees of freedom to detect many mis-identifications of latents, but the Figure 2 model has even more degrees of freedom, and its restrictive latent causal claims assist detection of additional inconsistencies.

We caution against thinking the nesting of the Figure 2 model within the Figure 1 model permits confident use of a χ^2^-difference test as testing just the constraints added (the coefficients given fixed values) in moving to the Figure 2 model. An ill-fitting Figure 1 model clearly reports evidence of problems beyond or without the added constraints – so a fitting Figure 1 model is a precondition for any such claim. If the less-restricted Figure 1 model is properly causally specified (despite containing some unnecessarily free latent covariances), then the χ^2^-difference test does indeed test the added constraints, but notice that the fit of the Figure 1 model does not assure us that the Figure 1 model actually is causally proper. The model may have managed to fit by choosing an incorrect compromise identity for a variable like η_3_, or incorrect identities for several latent variables. Hence the failure of the more restricted (Figure 2) model may, or may not, be signaling the improperness of even a fitting Figure 1 style model. The failure of a Figure 2 model might result from incorrect placement of null causal connections between some latent effects (so rearrangement of the latent effects could render the model proper) but the failure of the more restricted model might instead be reporting that some latent variables in the Figure 1 model were problematic, even if initially undetectably so.

Adding indicators clustered under specific latents, while retaining a saturated latent-level model like Figure 1, provides additional testing but it is testing that fails to cogently test whether the latents can be appropriately coordinated by latent-to-latent causal actions. A fitting Figure 1 style model with additional clustered indicators, provides evidence that only one latent underlies the clustered items, but this can be a Trojan horse surreptitiously sneaking in a latent like η_3B_ rather than a proper causally-connectable η_3A_ or η_3C_. More indicators (even in fitting Figure 1 style models) do not necessarily mean better latents, they mean more entrenched latents – where the entrenchment is provided by the indicators, with the possible sacrifice of appropriate latent-level causal connectivity.

Researchers locating latents via factor analysis have statistically/procedurally avoided stringent examination of whether the located latent factors are capable of engaging in causal actions at the latent level – and hence these researchers are prone to being rudely surprised when their “meaningful” latent factors fail to behave appropriately as latent causes and effects. Cross-over loadings leading from one latent to indicators of another latent exacerbate the problem of factor models morphing (via biased estimates) into χ^2^-fitting but causally-problematic models (see Hayduk & Glaser [27]). And using scales created by adding or averaging the values of multiple indicators make it more difficult to distinguish between latents such as η_3A,_ η_3B_, and η_3C_ because only the scale’s covariances appear in the data covariance matrix rather than the multiple indicators’ covariances. That makes the model less able to detect the type of inconsistency discussed above.

Collectively, these observations preclude making simple statements about what structural equation model tests test, even without enumerating the many additional features potentially leading to significant model ill fit – features such as violation of the presumed causal homogeneity of the cases, non-normality, or non-linearity. What remains undeniable is that any model with a fixed measurement error variance for either a single indicator, or best of multiple indicators, is more assuredly identified than the same model with a free (and potentially identification-disrupting) measurement error variance.

#### Specifying measurement error variances for single indicators and the best of multiple indicators

How then is a researcher to proceed? Our advice is to begin with a model that seems reasonable to you as researcher, and that is theoretically precise – a model like Figure 2 with constraints on the latent-level effects and constraints on the latent-to-indicator effects (whether this means using y5 as a single indicator of η_3A_, or y5 and y6 as multiple indicators of η_3B_, or y5 and y6 as single-indicators of both η_3C_ and η_3B_). This model should contain a fixed (usually nonzero) measurement error variance for each indicator having a 1.0 loading that specifies a scale for a latent – namely for the best (possibly the only) available indicator of each latent.

To obtain a fixed numerical measurement error variance, the researcher begins by carefully considering the latent level causal structure, to gain a clear sense of how each latent is expected to causally function with respect to the other modeled latents and with respect to specific imagined error variables like error-A, error-B, error-C and error-5 in Figure 3. The researcher explicitly considers how far the causal consequences of each specific imagined error would spread through the model. The researcher then seeks the best, or few best, indicators for each latent. “Best” here refers to the indicator most clearly reflecting the researcher’s desired meaning for each latent. For indicators obtained from questionnaires, the researcher should consider whether the respondents know themselves in ways that permit even truthful and uninhibited responses to causally originate in the values of the intended latent. The question wording, the context provided by other questions, and the available response options are all relevant to this assessment. The researcher should filter out questions having confusing or inappropriate wordings, likely misinterpretations, insufficient or unclear response options, and restricted or skewed response ranges.

There is no good reason to shade one’s measurement error variance assessment to be artificially small. Such a preference constitutes a bias against a latent like η_3A_ because using y5 as an indicator of η_3A_ requires cumulating more errors. But notice that the measurement error variance specification for y5 might justifiably use a *smaller* error variance specification than suggested by y5’s loading or reliability from prior factor analyses. Other researchers may have used y5 to locate η_3B_ via factor analysis (with additional indicators like y6) but that does not forbid the current researcher from using η_3C_ as their latent, which would require a *smaller* error variance on y5 than was observed in factor analysis. Measurement error is not something vaguely "out there", and it is *not* something reported exclusively by an item’s methodology. What is modeled as measurement error also depends crucially on the researcher’s theory assertions and theory postulations. What counts as measurement error is intimately tied to the current researcher’s theory requirements and intentions. (Does η_3A_, η_3B_, or η_3C_ belong in the theory?) Error variance specifications attend to theory consistency, not merely to indicator correlations.

For example, if η_3A_, η_3B_, and η_3C_ in Figure 3 each directly caused the severity of one medical symptom, the resultant correlated-symptoms would *not* warrant claiming there was only a single underlying latent cause. Diagnostic symptom-sets address a variety of clinical exigencies but may or may not be properly specified as multiple indicators having one common cause. Structural equation researchers must learn to beware administratively routine variables whose causal foundations are imprecise or even misconceived. Similarly, beware the term “breadth”. All latent variables, including factors, have no “breadth” (they only have a magnitude or value on a skinny number line) no matter how many indicators or effects they have. Adding indicators does not add breadth to the latent – it adds additional concern for the properness of the model’s causal specification. The quality of coefficient estimation will decline if indicators are causally misspecified as multiple indicators.

Both the measurement error variance assessments and the model’s latent structure should reflect any methodological concerns with the indicators, including methodological mess-ups. If a methodological mess-up causally influenced the data, appropriately including that mess-up as part of the latent-level causal model adds explained, not error, variance and would result in unbiased estimates of the other model coefficients [16:31, xix]. Consider how a researcher might address the causal consequences of having several indicators obtained by the same method. We hesitate to say the concern is for a “specific factor” or “method factor” because some people would presume the only reasonable way to address this would be by adopting a traditional factor approach. A superior procedure might be to obtain the best indicator for a method-latent by selecting an indicator using the method but whose substance was disconnected from the other modeled latents. That best method-indicator should scale the method-latent with a fixed 1.0 loading and be given a fixed measurement error variance (the variance arising from everything except the method’s variance). The effects of the method-latent on all the other relevant indicators should be constrained to be equal unless theory justifies why some indicators should display more method’s response than others. This results in only two coefficients to estimate – the variance of the method latent and the effect magnitude connecting the method-latent to all the relevant indicators – and adds model degrees of freedom due to the new indicator’s numerous new data covariances. Selecting the “best indicator” of the method latent, specifying a fixed measurement error variance for that best indicator, and considering possible causal connections between the method latent and the other modeled latents would do more to clarify the nature of the measurement-method’s causal nexus than would a knee-jerk reaction pretending that calling something a “methods factor” requires use of an ordinary “factor”.

Indeed, it may sometimes be possible to model two latents (one being the latent of interest, the other being a specific or “method-factor” latent) with only a single indicator if the two latents are clearly and substantially differentially embedded at the latent level of the model. Unfortunately, the required and forbidden latent level causal relationships of “methods-factors” seem insufficiently specified in existing theories, though we hope awareness of this modeling possibility encourages appropriate theory developments. Other methodological concerns might involve violation of the presumed independence of the latent-level errors in Figure 3, or an unmeasured cause of an intended latent also causing an indicator via mechanisms not currently in the model. These kinds of concerns can be addressed but require modeling tricks beyond what we can discuss here (see Chapter 7 of [15], or [16]).

Obtaining the specific numerical value to use as a fixed measurement error variance is often assisted by the researcher making their assessments as percents of the indicator’s variance that are likely to originate in the various potential causal sources of the indicator (the things paralleling error-5, or error-C, etc. in Figure 3). The researcher then obtains their specific asserted numerical value for the indicator’s fixed measurement error variance by multiplying the actual variance of the indicator by the sum of the percents assigned the features the researcher claims as error sources (all the things comprising error-5, or perhaps the things comprising both error-5 and error-C, and so on). Notice that the indicator's measurement error variance specification does not depend on how well the researcher expects the corresponding latent to be explained, or how well it explains other latents. An indicator that contains much measurement error, can correspond to a latent that explains much or little, or that is explained well or poorly by other latents – depending on the model and the operative real-world forces.

Those unaccustomed to making error variance assessments might familiarize themselves with the sensitivity of variance to the placement of extreme cases in a variable’s distribution by duplicating a variable from a data set (so the real data remains pristine) and using their favorite program to plot the variable’s distribution and calculate the variable’s variance when some cases’ values are changed, for example by: moving some values from below the mean to above the mean, taking central values and making them extreme, taking extreme values and making them central, moving many or all the cases’ values up, or randomly moving some cases’ values up and others down. Observing the distributional changes, and percentage changes in variance, that result from such artificial causal interventions assist in making more realistic error variance assessments. For example, if a variable has a skinny-tailed distribution, only a very few cases are likely to have obtained extreme values “in error” because only a portion of the already-few extreme cases are likely to have obtained those values erroneously. Assessments of error variances must respect the observed variable’s distribution.

Some researchers experience an urge to estimate as many measurement error variances as possible – thereby avoiding fixed measurement error variances. We recommend researchers curb this urge for the sake of theory precision. Being able to estimate a measurement error variance does not mean one should do this. Specifying a fixed measurement error variance for the best available indicator assists model identification (or over-identification), but this is *not* done because the researcher must do this for model identification. The fixed measurement error variance contributes to theoretical precision. Freeing the measurement error variance on the best available indicator amounts to succumbing to an estimation-invitation to theoretical imprecision. (The measurement error variance on a second indicator is typically left free because once the latent’s identity has been controlled by the best indicator’s specification, the second indicator’s free loading and measurement error variance provide an assessment of how good or poor that second-best indicator is at reflecting the latent specified via the best-indicator.) Even single indicators can have identified measurement error variances (for example, if the single-indicated latent also causes several other latents) but here also the researcher should demonstrate their commitment to theory by resisting estimation of the single-indicator’s measurement error variance.

Others feel an urge to estimate as many error variances as possible, to avoid those specifications potentially contributing to model failure. This inclines the researcher toward theory-imprecision merely to reduce the possibility that the data will speak against their theory. Making a theory imprecise does indeed make it more difficult for the data to detect problems in the theory – but that same imprecision makes it easier for the discipline to disregard the researcher’s work! Researchers using “lack of certainty” as an excuse to estimate the best indicator’s measurement error variance should be heard as theory bashing, theory belittling, or theory deficient – depending on whether their statement is made brutishly, snidely, or as an honest expression of incapacity as theorist. From the factor-analytic perspective, a fixed measurement error variance is non-conventional, though the extra theory-precision clearly supersedes factor-analytic tradition.

#### What if the theory-laden model fails to fit?

If a Figure 2 style model fails to fit the data according to χ^2^, this provides evidence that something has gone wrong. Unfortunately there is no generally-applicable procedure that can assuredly identify specifically which of the many potentially problematic things has gone awry. The best the researcher can do is report and respect the evidence of problems, and seek diagnostic signs pointing toward or away from specific possible problems.

The modification indices might suggest improving the fit by freeing the fixed measurement error variance on y5 (putatively the best available indicator), but this should *not* be done without a thorough reconsideration of the features discussed above. The corresponding "expected parameter change" statistic might suggest increasing or decreasing y5's fixed measurement error variance, where increases or decreases should be thought of as moving up or down among latents like η_3A_, η_3B_, and η_3C_. But remember that latents like η_3A_ might never be modelable if both y5 and y6 are used as multiple indicators in the model. Instead of freeing y5's measurement error variance at the behest of the modification index, the researcher might decide to drop y6 and thereby permit changing the fixed error variance on y5 to locate η_3A_. Or the researcher might decide to make y5 an indicator of η_3C_ and y6 an indicator of η_3B_ so that both y5 and y6 receive fixed measurement error variances, and the model contains two similar yet distinct η_3_ latents (η_3B_ and η_3C_). With y5 and y6 as single indicators of separate latents, the complex but theory-beneficial reconsiderations would focus on how theory could incorporate two slightly different versions of what previously had been incorrectly thought of as a single latent η_3_.

Or suppose a substantial modification index appeared for the covariance between the errors on y5 and y6. This might signal need for coordination between these errors, but error covariances are frequently inserted without sufficient consideration. Measurement error variables are routinely assumed to be independent of the latents in the model, and that renders the causal foundations of measurement error covariances entirely disconnected from the original latent theory. Consequently, freed measurement error covariances tend to become fudge-factors that provide fit without any theory justification. It is preferable to view a substantial modification index for an error covariance on indicators like y5 and y6 as indicating the constraints in some portion of the model are incompatible with the constraints specifying y5 and y6 as originating in the common cause η_3B_. Thus the researcher’s thought process returns to considering Figures 3 and 4 and the various ways of responding to possibilities like η_3A_ and η_3C_. This keeps any subsequent model modifications intimately connected to the researcher’s original theory. The researcher should report whatever model modifications are made at the behest of the modification indices because these are theory-focused model revisions.

Notice that modification indices are unable to directly call for inclusion of new latent variables, or for removal of improper even if biasedly-significant effects. Also notice that if the researcher had used a scale (created from y5 and y6) as the indicator for η_3_ that would further impair the ability of the modification indices and other diagnostics to prod consideration of y5 and y6 as indicators of separate latents. A substantial modification index connected to a scale should initiate substantive reconsideration of all the items comprising that scale.

In short, the diagnostic investigation should be oriented toward theory reconsideration and theory revision, with fit or fail as secondary to the theory legacy. The researcher should report any incorporated changes as theory modifications – and maybe even theory advances – but this is getting uncomfortably close to indirect data snooping that biases model testing. Fortunately, entertaining the possibility of new and differentially causally embedded latents is not as statistically odious as directly following large modification indices. The retheorizing provides a research contribution whether or not it results in a fitting model.

The only way a fixed measurement error variance on a single-indicator contributes to model ill fit, or has a substantial modification index, is if that measurement error variance would be "identified if freed". The modification index and expected parameter change statistics could clearly point to this style of problematic coefficient. In contrast, a fixed measurement error variance that would be underidentified (un-estimable) if freed does not contribute to model ill fit and will have a zero modification index even if the current fixed measurement error variance value is too small or too large. For example, if y5 was a single indicator and its error variance was not rendered identified by the latent level of the model, the fit and modification indices would be unable to signal a problematic error variance specification, or warn of the biases in the latent effect estimates that might arise from this. The amount of bias introduced by undetectable measurement error misspecification depends on many things, the most important being the magnitude of the misspecification. For example, if y5’s underidentified-if-freed measurement error variance was fixed at zero (thereby claiming no measurement error), the extent of the bias this introduces would depend on whether the true latent was η_3A_, η_3B_ , or η_3C_. If the true latent was η_3A_, the fixed zero value would be most-misspecified and the coefficient estimates most biased – even if undetectably biased.

This style of problem commonly appears when demographic variables like sex or age are assigned zero measurement error variance. There is clearly some measurement error variance in age – because age accumulates progressively even if measured in years. Measurement error in reported sex becomes more obvious if one considers some respondents as “reporting a wrong sex just for the fun of it,” or models where the latent effects of sex arise from the number of Y chromosomes rather than self-labeling or from genital appearance (which may have been “surgically assigned” just after birth, or reassigned later in life). The estimated effects of age and sex will be biased unless the appropriate measurement error is entered into the model – whether or not the omitted measurement error variance on age or sex results in noticeable ill fit or modification indices. Specifying a small non-zero measurement error variance for any single indicator (for age, sex, or whatever) is likely to provide less-biased estimates than an obviously-wrong specification of zero measurement error variance, but to consider this carefully one must again consider the causal forces preventing the indicator from precisely tracking the values of the intended latent variable. We empathize with those struggling to determine the amount of measurement error to specify, but we will rebuke anyone who pretends the difficulty justifies specifying zero measurement error variance – because that pretends the difficulty of the task justifies using an extreme and unjustified value (zero).

Fortunately, there is a relatively simple way to assess the consequences of specific fixed measurement error variances on single indicators whether or not they would be underidentified if freed. The strategy has been dubbed the “half and double” procedure, and was popularized by Hayduk ( [15], page 125; [16], page 28). The consequences of an incorrect measurement error variance assessment can be assessed by running a series of models, each altering one fixed error variance to first "half” and then “double" the original fixed value. Half the researcher’s best assessment of the measurement error variance makes the measurement error variance about as low as it might reasonably be (it is half way to the unreasonable value of zero), and double makes this about as high as it might reasonably be. For each run the researcher monitors the other coefficient estimates (especially those directly connected to the latent whose measurement quality is being tickled) to see how sensitive those estimates are to the alternative measurement error variance specifications. Any substantial variation in estimates warrants careful consideration and report because these particular coefficient estimates are especially sensitive to the researcher’s corresponding measurement error variance specification.

### Real examples

**Figure 5:**
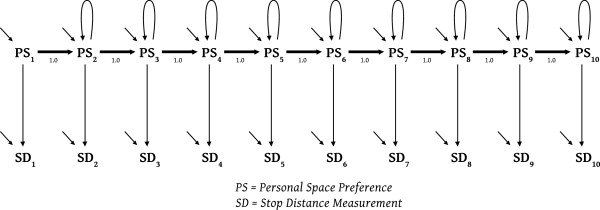
A real example.

Models employing single indicators with fixed measurement error variances emphasize theory, and precise theory does not lend itself to brief exposition, but we will try. The example in Figure 5 comes from Hayduk [37] and was chosen because it illustrates helpful-theory with minimal complexity among the indicators. The indicators are 10 measurements of subjects’ personal space preferences made as baseline (or control) measurements in an experiment whose treatments need not concern us. The indicator variables are distance measurements obtained by the stop-distance procedure in which the subject stops an approaching experimenter when the subject just becomes uncomfortable about the approacher’s closeness. The procedural similarity and clarity of the measurements, as well as multiple experimentally-controlled features [38] resulted in each indicator being given 3% measurement error variance. 

The background for the Figure 5 model is that back in 1985 a common factor model for the repeat personal space measurements failed convincingly. The measurements all used the same methodology, with the same participants, in the same baseline/control context, but the repeated measurements did NOT measure the same thing! A simplex model (a straight line of causal effects) fit via χ^2^, but that ordinary simplex model did not correspond to a comfortable or causally-understandable theory for these data. Nearly a decade passed before the fitting, understandable, and theory-helpful, loop-simplex model in Figure 5 was developed (Hayduk [37]). The 1.0 values connecting the latents in this model indicate that each subject’s spatial preference would have persisted perfectly from one measurement occasion to the next were it not for the structural disturbance terms and causal feedbacks modeled as self-causative loops at each successive measurement. This model illustrates a nearly-identical set of single indicators supporting a theoretically complex and somewhat unusual model structure – a model structure matching how the subjects’ brains acted causally in determining the subjects’ momentary spatial preferences. Additional single-indicator models permitted even closer parallels to causal neurological activity but this is not the place to discuss how the brain functions, or to explicate the statistical details of how causal-loops function, so we must be satisfied with referring the reader to Hayduk ( [37], [16] Chapter 3) for further discussion of the theory in the Figure 5 model.

**Figure 6:**
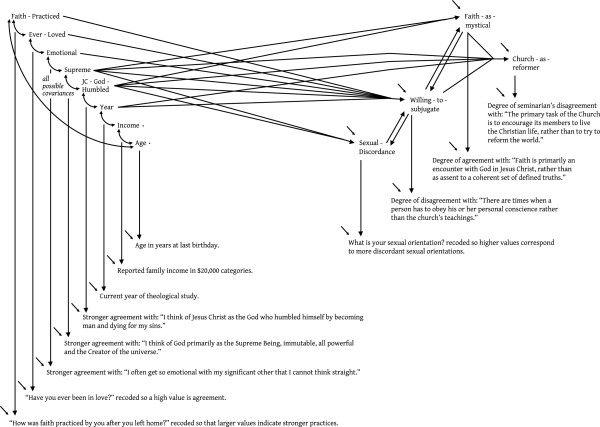
Another real example.

The example in Figure 6 was chosen because the latent level of the model is moderately complex – it has two touching reciprocal effects – that are cleanly estimated with single indicators assigned between 5 and 10% measurement error variance. This fitting (via χ^2^) model comes from an anonymous survey of Catholic seminary students, and the estimates tell some interesting stories, but we again refer the reader to the original publication for the details (Hayduk, Stratkotter & Rovers [39]). One especially relevant point is that a planned alternative model similar to Figure 6 was estimated in which two indicators (the indictors of Supreme and JC-God-Humbled) were modeled as arising from a single latent rather than two separate latents – much like trying to model y5 and y6 in Figure 3 as arising from η_3B_ rather than coming from separate latents – because it was unclear whether or not the seminarians’ responses arose from latents acting differently with respect to the other modeled latents. This common-cause model fit but showed clear diagnostic signs of model misspecification. That is, the seminarians’ agreement/disagreement with “I think of Jesus Christ as the God who humbled himself by becoming man and dying for my sins.” and “I think of God primarily as the Supreme Being, immutable, all powerful and the Creator of the universe.” were not tapping a single belief but were tapping two distinct latents that functioned somewhat causally-differently with respect to the other latents in the model. We chose this example because we expect some readers will find it surprising that indicators having such abstract yet seemingly-similar content could be clearly differentiated by a rather complex and difficult-to-estimate latent model – despite all the other latents also having only single indicators. This illustrates how latent level theory – in this case aided by planned diagnostics – can call for single indicators that differentiate between similar yet undeniably abstract latents. 

## Summary and discussion

The gist of the above is that each single indicator of a latent, and the best indicator from each set of multiple indicators, should be provided a fixed 1.0 “loading” and a fixed measurement error variance based on the researcher’s assessment of both the indicator’s methodology and the focal latent’s causal connections to the other latents. The fixed 1.0 loading scales the latent and the fixed measurement error variance assigns a theory-dictated identity or meaning to the latent. This is not done on the basis of what the researcher unerringly knows, but on the basis of what the researcher thinks s/he knows, so that the data via the estimates, testing, and diagnostics, speak to what the researcher thinks s/he knows. Any additional indicators believed to originate in the latent are given free loadings and free measurement error variances. While this description and our discussion focuses on reflective indicators, it should be clear that similar observations apply to both formative and reactive [40] indicators. The fundamental concern is for valid and precise latent-indicator representations no matter what style of measurement structure is involved.

Understanding that the latent variable absorbing and emitting latent-level effects must match the latent variable influencing that latent’s indicators reveals why multiple indicators located by factor analysis tend to fail when incorporated in structural equation models. Free factor correlations place no latent level constraints on the factors, and hence latent factors are permitted to become variables that are unable to function causally appropriately with respect to other latents. Even fitting factor models provide no assurance that the latent common factor causes of the indicators will function causally-appropriately with respect to other latents. Consequently, introducing latent level theoretical constraints often challenge the causal appropriateness of factors connected to multiple indicators. Researchers should hear saturated factor correlations and entirely free measurement error variances as shouting THEORY IMPRECISION regarding the latent-level causal structure.

The existence of multiple similarly worded indicators is no longer a license to squelch theory by saturating the latent level of the model with factor correlations, or by failing to assert a latent’s meaning with a fixed measurement error variance for the best of the multiple indicators. Focusing on single indicators, and designating the best of multiple indicators, encourages attention to each latent and indicator, and constitutes a call to theorize carefully. The identity of latents is not resolved by appealing to just the data – this unavoidably involves the researcher’s understandings and causal theory [41]. It is dubious to attempt “measurement” prior to “theory”, or factor analysis prior to full structural equation modeling, because there is no routine assurance that latents functioning as common causes of the indicators will assuredly function appropriately as causally-coordinateable latents.

If a full structural equation model fails and provides diagnostics questioning some second or weaker indicator, the researcher might drop that indicator but it would be preferable to retain the indicator by making it a single indicator of a similar yet theoretically distinct latent whose causal coordinations deserve explication. Much is also gained by using single indicators to incorporate multiple-regression-like control for potentially confounded variables. If the model requires control for sex, age, disease severity, number of friends, happiness, or belief in an after-life, the relevant control variables are latents because they likely contain measurement error. There seems little reason to require more than a single even if error-containing indicator for sex or age, but it is important to realize that the researcher could also statistically control for the other listed variables with single indicators. The researcher must decide whether a stronger research contribution would arise from using an additional indicator as a redundant multiple indicator of some currently-modeled latent, or by using that additional indicator to control for some causal mechanism currently omitted from the model. One does not need multiple indicators to locate a mechanism carrying a postulated effect, to extend a theory’s reach, or defend a theory’s claims by controlling some confounder. Hence the choice will often favor a single indicator controlling for some theory-relevant feature rather than multiply entrenching a particular latent.

Single indicators forcefully remind us that measurement is not separate from theory. Theoryphobes may consider single indicators too theory demanding, but researchers should think of single indicators as theory-encouraging and theory-invigorating. Single indicators challenge people to join the community of researchers, where one's constant environ is imperfect-knowing, and where detailed attention to theory and methodology are one's most trustworthy guides. Careful consideration of single indicators encourages a close coordination between the researcher’s thinking and their structural model, whether any specific latent ends up with one, two, more, or even no [16], direct indicators. When researchers place their understandings in their models, they hear their data speaking to them because it is their understandings that are being revised if the data urges model modification. Conscientious use of single, or the few best, indicators contributes to theory/model precision but it remains for the world to dictate whether the precise theory/model is valid or precisely wrong.

## Competing interests

The authors declare that they have no competing interests.

## Authors’ contributions

LAH participated in the SEMNET discussions during which the fundamental ideas behind this article were discussed, and he prepared the full first draft (including figures), and later draft revisions. LL initiated article preparation, and reviewed and suggested revisions to multiple drafts. Both authors have read and approved the final manuscript.

## Pre-publication history

The pre-publication history for this paper can be accessed here:

http://www.biomedcentral.com/1471-2288/12/159/prepub
